# Integrated genomic, transcriptomic, and epigenetic analyses identify a leukotriene synthesis-related M2 macrophage gene signature that predicts prognosis and treatment vulnerability in gliomas

**DOI:** 10.3389/fimmu.2022.970702

**Published:** 2022-09-08

**Authors:** Hang Ji, Zhihui Liu, Nan Wang, Jiaqi Jin, Jiheng Zhang, Jiawei Dong, Fang Wang, Xiuwei Yan, Qin Gong, Hongtao Zhao, Haogeng Sun, Yongzhe Li, Shaoshan Hu, Chao You

**Affiliations:** ^1^ Department of Neurosurgery, West China Hospital, Sichuan University, Chengdu, China; ^2^ Cancer Center, Department of Neurosurgery, Zhejiang Provincial People’s Hospital, Affiliated People’s Hospital, Hangzhou Medical College, Hangzhou, China; ^3^ Department of Neurosurgery, Second Affiliated Hospital of Harbin Medical University, Harbin, China; ^4^ School of Life Sciences, Nanjing University, Nanjing, China

**Keywords:** lower-grade glioma, glioblastoma, PI3K/Akt signaling pathway, leukotrienes, M2 macrophage, tumor microenvironment

## Abstract

The pathological implications of tumor-associated macrophages in the glioma microenvironment have been highlighted, while there lacks a gene signature to characterize the functional status and clinical implications of these cells. Comprehensive bioinformatics approaches were employed to develop an M2 macrophage-associated gene signature at bulk-tumor and single-cell levels and explore immunological and metabolic features. Consequently, the PI3K pathway and fatty acid metabolism were correlated with the M2 fraction. Further distilling the pathway members resulted in a leukotriene synthesis-related gene signature (Macro index), including PIK3R5, PIK3R6, ALOX5, ALOX5AP, and ALOX15B, that was primarily expressed by monocytes/macrophages. Increased Macro index predicted IL13-induced macrophages, and was associated with T-cell dysfunction at both transcriptional and epigenetic levels and predicted an unfavorable outcome. Besides, the Macro index was proportional with PAI1 at the protein level, with high levels of the latter suggesting a decreased progression-free interval of glioblastoma. Notably, the monocytes/macrophages in the glioma environment contribute to the expression of immune checkpoints and the Macro index predicts glioma responsiveness to anti-PD1 treatment. Together, our study proposed a leukotriene synthesis-related M2 macrophage gene signature, which may provide insights into the role of these cells in the glioma microenvironment and facilitate individually tailored therapeutic strategies for the disease.

## Introduction

Gliomas represent the most common types of brain malignancy that are characterized by high morbidity and mortality. This group of heterogeneous tumors accounts for approximately 80% of primary brain malignancies (1–3), and includes lower-grade glioma (LGG) that comprising WHO II and III diffuse low grade glioma and intermediate grade glioma and WHO IV GBM with some differences in aetiology, histology, and molecular underpinnings (1, 4, 5). Research achievements in gliomas culminate in the proposal of classification schemes and molecular biomarkers with prognostic and therapeutic implications. Histologically, gliomas are classified as astrocytomas, oligodendrogliomas, and mixed oligodendrogliomas based on the morphological characteristics of the tumor cells (1). The addition of anaplasia features (mitotic activity, microvascular proliferation, and necrosis) led to the WHO tumor grade for glioma which is indicative of malignant degrees (6). At the molecular pathogenesis level, the status of isocitrate dehydrogenase (IDH) gene mutation and chromosome arm 1p19q deletion defines three types of invasive gliomas: IDH-mutant with 1p19q co-deleted, IDH mutant with 1p19q non-co-deleted, and IDH wild-type glioma, greatly advancing our knowledge of the etiology (1, 7, 8). In addition, transcriptome subtyping of IDH wild-type glioblastoma (GBM), including proneuronal, mesenchymal and classical, delineated an insightful theoretical basis for the evolution of GBM subtypes and provided vital evidence that NF1expressing macrophage/macroglia facilitates the transformation of GBM subtype towards worse (9, 10). Nevertheless, glioma, especially malignant glioma, remains treatment-resistance (11). The mainstay of treatments, including invasive surgery combined with radiotherapy and alkylating agents, as well as newly thriving tumor treating fields (TTF), are far from achieving satisfactory improvements for patients with malignant glioma. Immune checkpoint blockade (ICB) therapy, which is a landmark in several types of tumors, is still dismally effective in the treatment of GBM (12–14). Therefore, there remains an urgent need to explore key molecular mechanisms in the pathological process of glioma and to develop new molecular biomarkers for individually tailored strategies.

The tumor microenvironment (TME) instigated by tumor cells is a complex, active ecosystem in which tumor-associated macrophages (TAMs) have content and functional superiority over other non-tumor cells (15, 16). TAMs in glioma TME indicate two types of cells of different origins: intrinsic microglia and blood-derived monocytes/macrophages, with functional consistency when interacting with glioma cells (17–19). The TAMs in TME are dogmatically defined as pro-tumoral M2 phenotypes and anti-tumoral M1 phenotypes, and this dichotomy is overly simplistic and a much broader repertoire of the polarization of macrophages lays the foundation of difficulties in designing therapies targeting M2 macrophages (20, 21). Transcriptome-based studies further defined the M2 phenotype at the gene expression level and identified the expression and functional characteristics of macrophage polarization mediated by various stimulators (22, 23). However, due to the immunological specificity of the central nervous system, the phenotype of TAMs in glioma TME remains loosely defined. Overall, TAMs play a paramount role in promoting angiogenesis, tumor invasion, and impeding antitumor immunity, the latter being closely related to the local chronic inflammation mediated by eicosanoid derivatives (15, 24, 25). TAMs derived and differentiated from blood precursor cells have theoretically a complete set of eicosanoid-metabolizing enzymes, and previous studies have emphasized the role of prostaglandins such as PGE_2_ in remodeling the TME, while the LOX pathway and leukotrienes are overlooked. Moreover, the role of the oncogenic PI3K/Akt signaling pathway, one of the hallmarks of GBM, in the regulation and integration of tumor metabolism, and thus in promoting tumor development, is gaining increasing attention (26, 27). In addition to glycolysis, the interaction of PI3K/Akt signaling with eicosanoid metabolism in TAMs is less understood, and exploring its pathological and clinical significance and association with TAMs phenotype may lead to novel therapeutic targets.

In this study, we explored the signaling pathways and gene expression associated with the fraction and phenotype of TAMs in glioma TME through correlation and network analysis based on multi-omics data from multiple glioma cohorts. We identified a gene signature linking to TAMs (Macro index) and validated their association with the functional status of TAMs, immune function, prognosis, and immunotherapy efficacy at the bulk-tumor and single-cell levels. These results may open attractive avenues for designing novel glioma therapy since leukotriene synthesis plays a vital role in glioma TME, and provide an effective mRNA metric for characterizing alternatively activated TAMs for glioma.

## Materials and methods

### Sample collection

Multiple glioma datasets from WHO grade II to IV were included in this study. Of these, the integrated mRNA expression profile (n = 702), single nucleotide polymorphisms (SNP) (n = 825), copy number variation (CNV) (n = 1122), and methylation (n = 685) profiles were retrieved from the UCSC Xena portal, and clinical phenotypes, including transcriptome subtype, were summarized by Ceccarelli et al. (28). Other glioma expression profiles with corresponding demographics, including CGGA693, CGGA325, CGGA301, Ducray, Gravendeel, Joo, Nutt, and Kamoun were retrieved from the CGGA and the GlioVis data portal (29–32). Bulk-tumor mRNA sequencing data were TPM normalized for further analysis. The single-cell transcriptome profile was retrieved from the TISCH database (Glioma GSE131928 10X) (33, 34).

### Consensus clustering and sample selection

Three *in silico* algorithms were performed to infer the immune infiltration, including CIBERSORT, QUANTISEQ, and XCELL (35–38). The CIBERSORT-derived immune infiltration fraction was used for consensus clustering. Samples with a p-value > 0.05 and cells with a fraction of 0 in over half of the samples were excluded. Consensus clustering was employed to stratify the immune infiltration fraction matrix (39). The maximum number of clusters was set to 6, the clustering algorithm was ‘PAM’, and the distance was set to ‘Pearson’. The optimal number of clusters was determined by the proportion of ambiguous clusters (PAC) method. Identification of core members of each cluster was based on the R packages ‘cluster’ and ‘vegan’. Samples with silhouette width ranked top 75% were included.

### Identifying signaling pathways and gene signature associated with M2 fractions

Gene sets of the HALLMARK (n = 50), BIOCARTA (n = 292), and PID (n = 196) were retrieved from the MSigDB database (v7.5.1), and the GSEA software (v4.2.3) and ssGSEA algorithms were employed to assess the pathway activity (40–42). The correlation between signaling pathways and gene expression with M2 fraction was evaluated by calculating the regression coefficient using a multivariate regression model. Logistic regression analysis was performed to evaluate the correlation of SNP and somatic copy number alteration (SCNA) with the M2 fraction. The vif value of each independent variable was adjusted to within 5 using the R function ‘step’ to avoid the potential interactions between independent variables. The differentially expressed genes (DEGs) were calculated using the R packages ‘limma’ and ‘edgeR’ (43, 44). Functional enrichment analysis was performed using the web tool Metascape (45, 46). We defined the Macro index as the average log2 transformed TPM value of PIK3R5, PIK3R6, ALOX5, ALOX5AP, and ALOX15B. The ranked gene list for GSEA was sorted according to logFC values or Spearman rho of genes of interest with Macro index.

### scRNA-seq data analysis

The expression of immune checkpoints at the single-cell scape in multiple datasets was integrated using the webtool TISCH. The R package ‘Seurat’ was employed for the management of sample quality control, normalization, data dimensionality reduction, clustering, and re-clustering of the scRNA-seq expression profile (GSE131928 10X) (47). Identification of the cell identity was based on the CellMarker database and signature genes summarized by Neftel et al. (48). The expression profile of the Mono/Macro subcluster was extracted, and Mono/Macro cells were split into Macro index-high and -low groups. DEGs (Macro index-high vs. -low) were calculated using ‘Seurat’, and transcription factor enrichment analysis was performed using the web tool Metascape based on the TRRUST database (49). Functional enrichment analysis was performed using Cytoscape plugins ‘Bingo’ and ‘EnrichmentMap’. To evaluate the association between the Macro index and function state of TAMs, the top and bottom 150 DEGs of macrophages (**Supplementary File 1**) under different culture conditions were used as the corresponding gene signatures, including glucocorticoids (GC), IL-10, IL-13, IL-4, and PGE_2_ (23).

### Evaluating immunological characteristics and potential ICI responsiveness

The anti-tumor immune response was conceptually divided into 7 stepwise events, including step1. Cancer antigen releasing, step2. Cancer antigen presentation, step3. T cell priming and activation, step4. Trafficking of immune cells to tumor, step5. Infiltration of immune cells to tumor, step6. Tumor call recognition, and step7. Tumor cell killing (50). The activity of each stepwise event was assessed using the webtool TIP. Macrophage functional status gene signature was defined as the top 150 up- or down-regulated genes in macrophages cultured under specific conditions for 72h (**Supplementary File 1**). T cell dysfunction gene signatures were generated based on the shRNA screen and have been summarized by Jiang et al. (51). Positive hit genes were defined as the upregulated genes and negative hit genes were the downregulated genes. The Spearman rho between Macro index and positive or negative hit genes were used as input of ROC analysis for evaluating the concordance of Macro index with macrophage functional status and T-cell dysfunction. Besides, sample responsiveness to immune checkpoint inhibitors (ICI) was predicted using the TIDE algorithm (51). The Submap algorithm was also employed to classify the sensitivity of glioma samples to ICI treatment, as referenced by a cutaneous melanoma cohort receiving PD-1 and CTLA4 inhibitors (52, 53). Another uroepithelial tumor cohort with a corresponding response to PD-L1 inhibitors was introduced to this study (54). Samples were split into Macro index-high and -low groups following the above methods. The composition of patients with progressive disease (PD), stable disease (SD), partial response (PR), and complete response (CR) included in the two groups was compared.

### Statistics

All statistics were performed using R software (v4.1.1). Log-rank tests and the Cox-ph model were used to classify survival differences. Two-tailed Wilcoxon test was employed to compare the difference in the immune infiltration fraction, ssGSEA scores of signaling pathways, and gene expression. The correlation between gene expression, mutation, or SCNA with M2 fraction was assessed using constructing multivariate and logistic regression models or Spearman correlation tests. In the regression analysis, two or more significant regression coefficients with the same positive or negative sign were considered statistically significant. Fisher’s exact test was employed to compare the composition ratios. In the absence of a specific statement, a p-value < 0.05 was considered significant.

## Results

### Combined M2 macrophage fraction and histology defined three groups of gliomas

**Figure 1:**
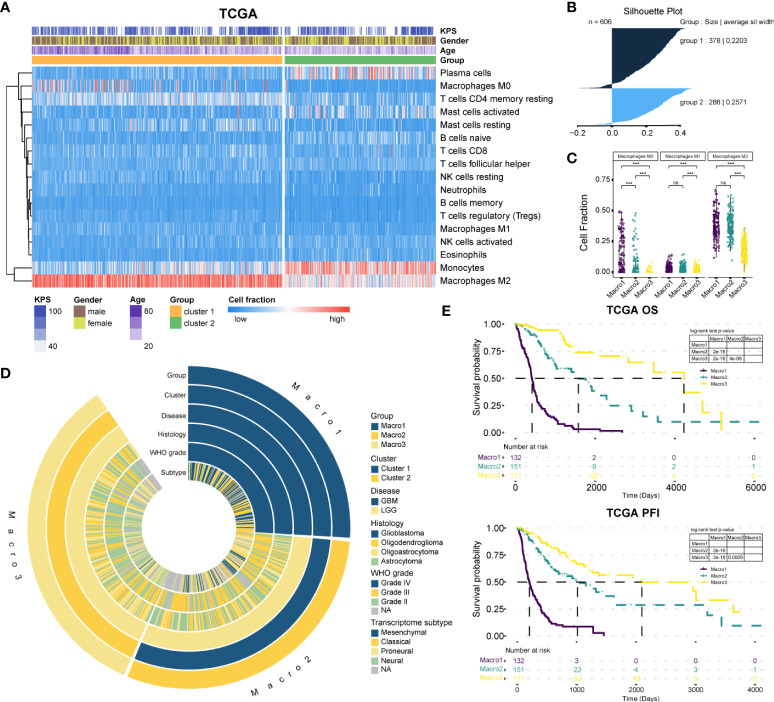
The classification of gliomas. **(A)** The abundance of estimated immune infiltration of glioma clusters. Five types of cells including dendritic cells activated, dendritic cells resting, T cells CD4 memory activated, T cells CD4 naïve, and T cells gamma delta were excluded for low content. **(B)** The silhouette width for the selection of core samples of each cluster. **(C)** Comparison of the fraction of macrophages between glioma groups. **(D)** Association of the glioma groups with prevalent clinicopathological biomarkers. **(E)** The survival differences between glioma groups. ***p < 0.001. ns, non significant.

First, we re-clustered LGG and GBM samples based on M2 fractions estimated by CIBERSORT, and two clusters were identified by the Consensus cluster (**Figures 1A**, **S1A-B**). The cluster with significantly increased M2 macrophage fraction was defined as cluster 1, and the other cluster 2. Ranking the silhouette widths of samples in descending order, the top 75% of samples were selected from each cluster (cluster 1, n = 283; cluster 2, n = 171) for further analysis (**Figure 1B**). Cluster 1 of the TCGA cohort is comprised of 132 GBM and 151 LGG, which differ significantly in pathogenesis. Thereafter, cluster 1 was further divided into two subgroups, namely Macro1 (GBM) and Macro2 (LGG). On this basis, the TCGA glioma samples were split into three subgroups (Macro1, n = 132, GBM; Macro2, n = 151, LGG; and Macro3, n = 171, LGG), with Macro1 and Macro2 containing comparable M2 macrophages, and Macro3 the least (**Figures 1C**, **S1C**). Following the same procedure, we identified cluster 1 and cluster 2 in the Rembrandt and CGGA693 cohorts. Thus, the GBM samples in cluster 1, the LGG in cluster 1, and the LGG in cluster 2 were defined as Macro 1, Macro 2, and Macro 3, respectively (**Supplementary File 2**).

The clinical features between groups were exhibited (**Figure 1D** and **Table 1**). The transition of transcriptome subtypes from Classical (CL) and Mesenchymal (ME) dominance of Macro1 to Proneural (PN) and Neural (NE) dominance of Macro3 was found. Although Macro2 and Macro3 were both LGG, Macro2 had an increased proportion of WHO grade III tumors (58.94%, fisher’s exact p = 0.00004) (**Table 1**). As expected, Macro1, Macro2, and Macro3 differed significantly in OS and PFI, where Macro1 had the most unfavorable prognosis (**Figures 1E**, **S1D**).

**Table 1:** Comparison of clinical features between subtypes.

Term	Macro1	Macro2	Macro3	P value
**Cohort**	**TCGA**			
Age	59.28 ( ± 14.02)	45.95 ( ± 13.47)	40.11 ( ± 12.57)	< 2.2E-16
**Gender**				
Male	88 (66.67%)	85 (56.29%)	98 (57.31%)	
Female	44 (33.33%)	66 (43.71%)	73 (42.69%)	0.15
**Histology**				
Astrocytoma		61 (40.40%)	44 (25.73%)	
Oligoastrocytoma		33 (21.85%)	55 (32.16%)	
Oligodendroglioma		42 (27.81%)	58 (33.92%)	
Glioblastoma	132 (100%)			0.0097^a^
**WHO grade**				
WHO II		47 (31.13%)	93 (54.39%)	
WHO III		89 (58.94%)	64 (37.43%)	
WHO IV	132 (100%)			3.732E-05^a^
**Transcriptome subtype**				
Classic	40 (30.30%)	21 (13.91%)	2 (1.17%)	
Neural	6 (4.55%)	12 (7.95%)	58 (33.92%)	
Proneural	16 (12.12%)	49 (32.45%)	70 (40.94%)	
Mesenchymal	57 (43.18%)	20 (13.25%)	2 (1.17%)	< 2.2E-16
**Cohort**	**Rembrandt**			
**Age**	55-59	40-44	35-39	
**Gender**				
Male	62 (54.39%)	36 (59.02%)	41 (50%)	
Female	37 (32.46%)	12 (19.67%)	32 (39.02%)	0.1116
**Histology**				
Astrocytoma		23 (37.70%)	12 (14.63%)	
Oligodendroglioma		12 (19.67%)	31 (37.80%)	
Mixed		2 (3.28%)	1 (1.22%)	
Glioblastoma	114 (100%)			0.001111^a^
**WHO grade**				
WHO II		23 (37.70%)	46 (56.10%)	
WHO III		30 (49.18%)	22 (26.83%)	
WHO IV	114 (100%)			0.009657^a^
**Cohort**	**CGGA693**			
**Age**	48.39 ( ± 13.49)	38.26 ( ± 10.13)	40.11 ( ± 9.25)	2.2E-16
**Gender**				
Male	58 (61.70%)	64 (60.38%)	21 (58.33%)	
Female	36 (38.30%)	42 (39.62%)	15 (41.67%)	0.8638
**Histology**				
Astrocytoma		34 (32.08%)	10 (27.78%)	
Anaplastic astrocytoma		53 (50%)	12 (33.33%)	
Oligodendroglioma		4 (3.77%)	1 (2.78%)	
Anaplastic oligodendroglioma		13 (12.26%)	12 (33.33%)	
Anaplastic oligoastrocytoma		2 (1.87%)	1 (2.78%)	
Glioblastoma	94 (100%)			0.05901^a^
**WHO grade**				
WHO II		38 (35.85%)	11 (30.56%)	
WHO III		68 (64.15%)	25 (69.44%)	
WHO IV	94 (100%)			0.6858^a^

a Comparison between Macro2 and Macro3.

### Signaling pathways correlated with M2 fractions

**Figure 2:**
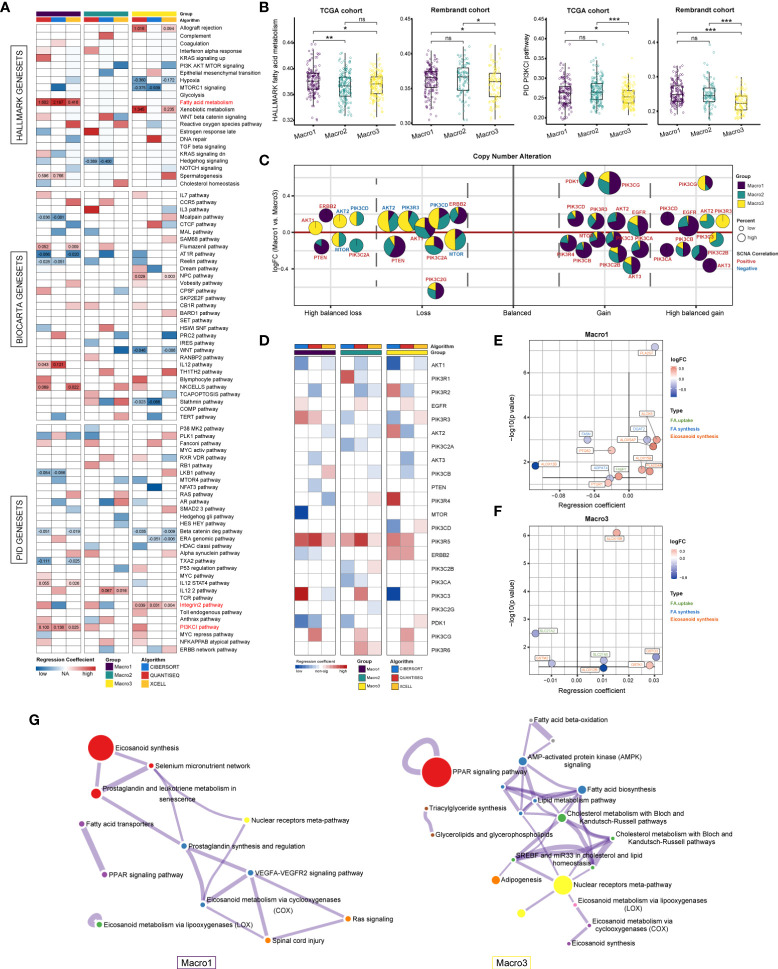
Resolving signaling pathways correlated with M2 fraction using multivariate regression analysis. **(A)** Correlation between M2 fractions and the 538 HALLMARK, BIOCARTA, and PID signaling pathways. Regression coefficients that were significant in at least two independent algorithms were marked. **(B)** Comparison of the ssGSEA score of the PID PI3KCI pathway and HALLMARK FA metabolism between groups. **(C)** Evaluation of the correlation between genes with significant copy number variants in the PI3K pathway and M2 fraction based on logistic regression. The colors represent the regression coefficients between a certain type of SCNA of a gene and the M2 fraction, with red being positive and blue being negative. The size of the bubble represents the frequency of SCNA. The area of the sector indicates the distribution of this type of SCNA of a gene in each group. **(D)** Assessing the correlation between the mRNA expression of PI3K pathway members and M2 fraction using multivariate linear regression. We determined results with significant and consistently positive and negative regression coefficients in two or more algorithms were statistically significant. **(E, F)** The correlation between FA metabolism genes and M2 fraction in Macro1 and Macro3. Colors represent the log2FC (Macro1 vs. Macro3). **(G)** Functional enrichment analysis of FA metabolic genes that are significantly associated with the M2 fraction in each group. *p < 0.05, **p < 0.01, ***p < 0.001. ns, non significant.

Next, we explored the signaling pathways affecting the M2 fraction. The gene expression profiles were converted into the HALLMARK, BIOCARTA, and PID signaling pathway matrices using the ssGSEA algorithm. Consequently, the FA metabolism, PI3KCI pathway, and integrin2 pathways were significantly correlated with the fraction of M2 macrophage in Macro1 and Macro3 (**Figure 2A**). Consistently, Macro1 had increased ssGSEA scores of PI3K signaling pathway and FA metabolism than Macro3 (**Figure 2B**). Besides, samples were also split into high, median, and low groups based on the M2 fraction, and the ssGSEA scores of the PI3K pathway and FA metabolism were increased in the macrophage-high group in Macro1 (**Figure S2A**), corroborating an association between the PI3K pathway and FA metabolism with the M2 macrophage. To further demonstrated the correlation between the PI3K pathway and M2 macrophage, members of the PI3K pathway were collected by massive literature search (**Supplementary File 3**). The mutation frequency of PTEN, PIK3R1, PIK3CA, and EGFR differed significantly between groups (Fisher’s exact test p < 0.0001), and the mutation of PTEN and EGFR was correlated with M2 fraction (**Figures S2B, S2C**). As expected, the frequency of SCNA in EGFR and PTEN decreased from Macro1 to Macro3 (**Figure S2D**), in line with the pro-tumoral role of dysregulated PI3K pathway. We systemically screened genes with significant differences in SCNA frequency between groups (Fisher’s exact p < 0.05) and exhibited the association between SCNA and M2 fraction (**Figure S2E**). For example, the M2 fraction was positively correlated with copy number gain of EGFR, AKT2, and PIK3CA and copy number loss of PTEN, AKT1, and ERBB2, while negatively associated with copy number loss of AKT2 and MTOR. The association between gene SCNA, expression, and their correlation with M2 fraction was summarized (**Figure 2C**). We found that copy number gain events that were positively associated with the M2 fraction tended to enrich in Macro1, while copy number loss events negatively associated with M2 tended to enrich in Macro3, indicating differential molecular mechanisms associated with the M2 fraction. In terms of gene expression, PIK3R5 was positively correlated with the M2 fraction across groups and AKT1 was negatively correlated with M2 in Macro1 and Macro3 (**Figure 2D**). Besides, the association between several genes and M2 was group-specific, such as PIK3R3, PIK3C2A, ERBB2, and PIK3CA.

Rewired FA metabolism also plays a vital role in remodeling the TME, which has been underestimated previously. We collected and exhibited the expression of genes involved in the *de novo* FA synthesis, FA uptake, and eicosanoid metabolism between groups (**Supplementary File 3**). As a result, Macro1 had significantly downregulated genes involved in the *de novo* FA synthesis, particularly ACACA, FASN, SCD, and SREBF1/2, and upregulated genes involved in FA uptake, such as CD36, SLC27A3, FABP5, and FABP7 (**Figures S3A, S3B**), while the opposite was true for Macro3, possibly indicating the heterogenous FA sources between glioma groups. Since eicosanoid metabolism as a branch of FA metabolism is involved in the production of many inflammatory mediators, we also exhibited the differences in eicosanoid metabolic-related gene expression between groups (**Figure S3C**). The expression of the PLA family, which generates AA through hydrolysis, differs between groups. For example, the expression of PLA2G4A, PLA2G2A, and PLA2G5 was significantly increased in Macro1, while the opposite was true for PLA2G6, PLA2G12A, and PLA2G4C. In terms of FA metabolic genes significantly associated with M2 fractions, a comparable proportion was found in genes involved in eicosanoid metabolism between groups (Macro1 8/12, Macro2 8/11, and Macro3 6/8), higher than that of FA synthesis and uptake (**Figure S3D**), suggesting that the eicosanoids metabolism was indeed related to the abundance of TAMs. Notably, we exhibited the types of these genes, their expression, and their regression coefficients with the M2 fraction. In Macro1, genes including ALOX5, ALOX5AP, and ALOX15B were upregulated and positively correlated with M2, and ALOX12B seemed to be a negative regulator that has been downregulated (**Figure 2E**). Interestingly, PTGS2, an inducible COX enzyme, was negatively correlated with the M2 fraction. There were few intersections of genes correlated with M2 in Macro1 and Macro3, except for ALOX15B/12B (**Figure 2F**), possibly indicating a functional transition of M2 macrophages in different classes of gliomas, with macrophages relying on ALOX15B for leukotriene synthesis in Macro3, which represents the lower grade, and ALOX15B and ALOX5/ALOX5AP in the higher grade. From a holistic perspective, functional enrichment analysis found that FA-metabolism associated genes upregulated in Macro1 were significantly enriched in leukotriene (ALOX5/ALOX15B/DPEP1/PTGR1) and prostaglandin (PLA2G4A/PLA2G5/PTGS1/PTGS2/PTGR1) synthesis, while in Macro3, only PTGDS, PLA2G6, and PNPLA3 were involved in the eicosanoid metabolism (**Figure 2G**), suggesting that genes involved in leukotriene synthesis were remarkably altered between low- and high-grade glioma.

### The intersection of PI3K signaling and FA metabolism defines M2 and leukotriene synthesis-related gene signature

**Figure 3:**
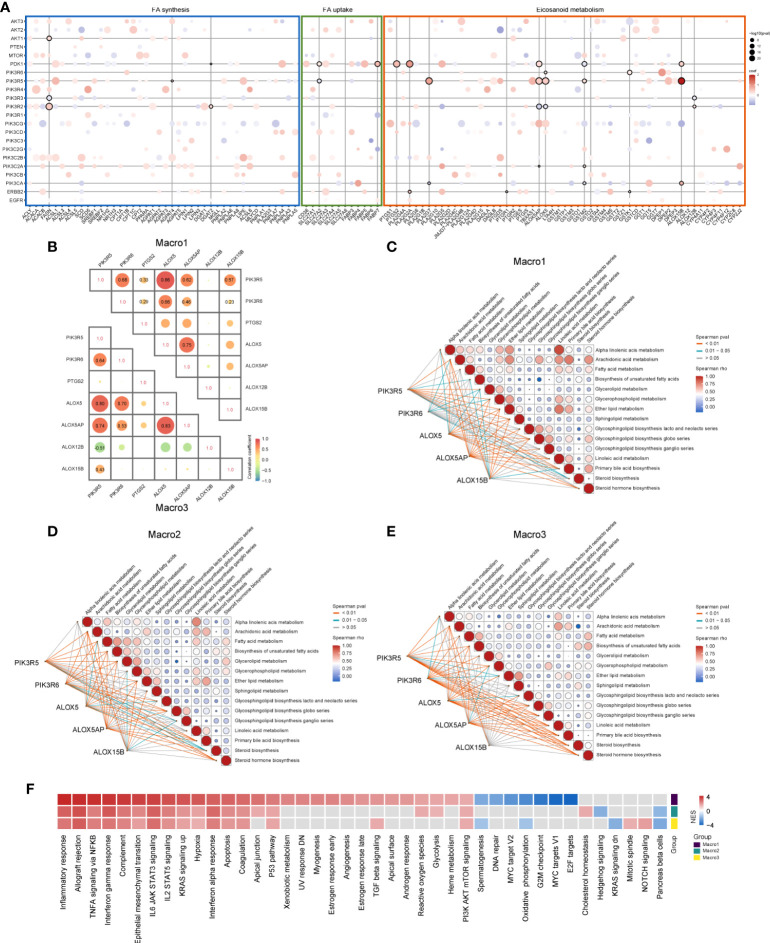
Association of PI3K signaling pathway members with FA metabolic genes that significantly correlated with M2 fraction. **(A)** The correlation between PI3K pathway members and FA metabolic genes was evaluated using a multivariate regression model at the significance level of p < 0.0001. Bubbles indicate that the regression coefficients of the two are significant, red indicates positive regression coefficients and blue negative. Horizontal or vertical lines mark PI3K members or FA metabolic genes that are significantly correlated with the M2 fraction. **(B)** Spearman correlation analysis of candidate genes that were likely to form a gene network. **(C-E)** Correlation of PIK3R5/6, ALOX5/5AP/15B, and KEGG lipid metabolism-related signaling pathways in each group. **(F)** GSEA analysis determines signaling pathways that affect the M2-related gene network based on the HALLMARK gene sets. The input pre-ranked gene list is a ranked list of genes determined by the Spearman rho of all human genes with the Macro index.

Several studies revealed the interaction between the PI3K signaling and FA metabolism (24, 25), we, therefore, addressed such association in glioma. The correlation of PI3K pathway members with FA metabolism-related genes was calculated using a multivariate linear regression model and genes that were significantly associated with M2 fraction were of particular interest. Genes significantly correlated with M2-related eicosanoid metabolism genes were mainly those encoding different subunits of PI3K (p < 0.0001) (**Figure 3A**). For instance, PIK3R5 was positively correlated with leukotriene metabolic genes like ALOX5, ALOX5AP, and ALOX15B. In addition to leukotriene metabolic genes, PDK1 was also correlated with PTGS2, which was shown to be negatively correlated with the M2 fraction. As Macro2 was a subset of LGG with increased M2 fraction when corrected for WHO grade, histology, and transcriptome subtype, the increased expression of PIK3R6 remained significant (**Figures S4A-C**). Besides, Spearman analysis showed a sparse association of PTGS2 and ALOX12B with PIK3R5/6, ALOX5/5AP/15B (**Figure 3B**). Therefore, we proposed that PIK3R5/6, ALOX5/5AP/15B comprised of an M2-related gene network associated with leukotriene metabolism (**Figure S5A**). In evaluating the association of these genes with FA metabolism pathways, we found that PIK3R6 was correlated with only a few pathways, including AA and glycerol metabolism, in Macro1, but with most FA metabolism pathways in Macro3 (**Figures 3C-E**), suggesting different levels of involvement of PIK3R6 in FA metabolism between groups. The opposite was seen in the correlation of ALOX15B with FA metabolism and immune pathways (**Figures 3C-E**, **S5B-E**). Thereafter, we defined the average expression of PIK3R5/6, ALOX5/5AP/15B as an mRNA metric (Macro index), which was significantly associated with the M2 fraction (**Figure S5F**). GSEA found that inflammation-related signaling pathways such as inflammatory response, allograft rejection, and TNFA signaling were correlated with Macro index across groups (**Figure 3F**), while the correlation between signaling pathways such as angiogenesis, TGFB, glycolysis, etc. and Macro index was differentiated. Together, these results proposed an M2 and leukotriene synthesis-associated gene signature, and the differential regulation mechanisms for this network among glioma groups.

### Differences in anti-tumor immune responses in glioma groups and the association with Macro index

**Figure 4:**
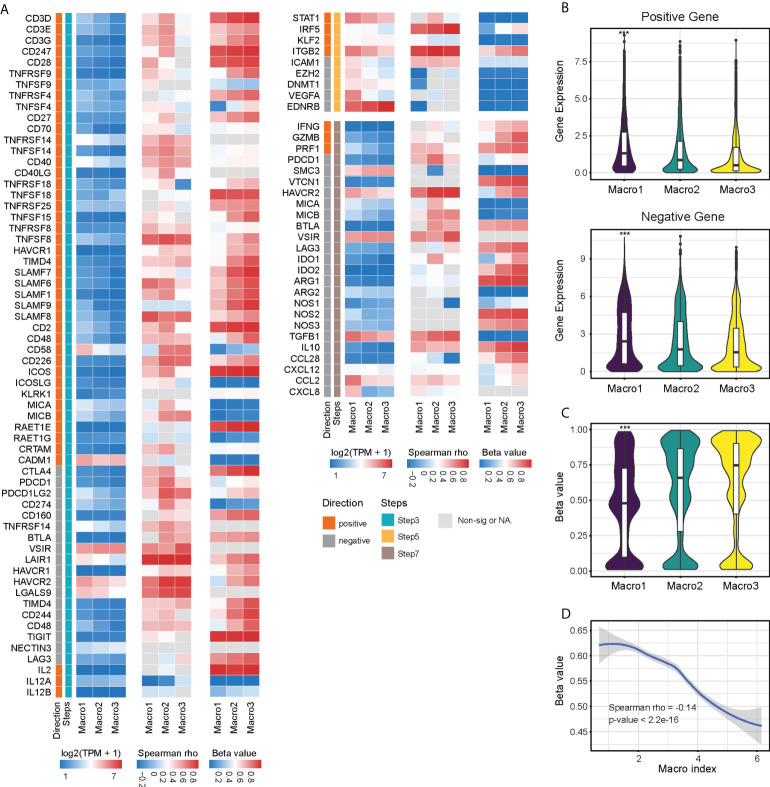
Immunological characteristics associated with Macro index-based groups. **(A)** Expression, methylation of immune-related genes and correlation with Macro index. Genes assigned to the TIP step3 (T cell priming and activation), step5 (infiltration of immune cells into cancer), and step7 (cancer cell killing) were included. **(B)** The overall expression of immune-activating and immune-suppressing genes in each group. **(C)** The overall methylation levels of these genes in each group. **(D)** Spearman correlation analysis of the Macro index with the overall gene methylation level. ***p < 0.001.

The anti-tumor immune response is critical in influencing tumor outcome and has been artificially defined as several step-wise events for quantitative assessment. In general, Macro1 scored higher in the recruitment of immune cells but was less active in T-cell activation and tumor cell killing than Macro2 and Macro3 (**Figure S6A**). We enumerated the expression and methylation of genes involved in T-cell activation (step3), immune cell infiltration into tumors (step5), and tumor cell killing (step7), and found that most of the differences in gene expression converged on their methylation levels (**Figure 4A**). However, there were exceptions, for example, the methylation levels of EZH2 did not differ significantly between groups and its differential expression may be regulated by factors associated with the Macro index (**Figure S6B**). The overall expression of genes that play either positive or negative roles in steps 3, 5, and 7 of the anti-tumor immune response were significantly increased in Macro1 (**Figure 4B**), suggesting that the activated immune response in Macro1 was accompanied by enhanced inhibitory mechanisms. The overall methylation level of the genes between groups was opposite to their expression level (**Figure 4C**), suggesting a role for methylation in regulating the immune response, and the Macro index was significantly negatively correlated with gene methylation level (**Figure 4D**). Similarities were found between the association of Macro index with immune responses in each group, positively correlating with immune cell recruitment but negatively correlating with T cell activation and tumor cell killing (**Figures S6C, D**), suggesting an active but ‘ineffective’ immune response.

### The association between the Macro index and the functional state of TAMs

**Figure 5:**
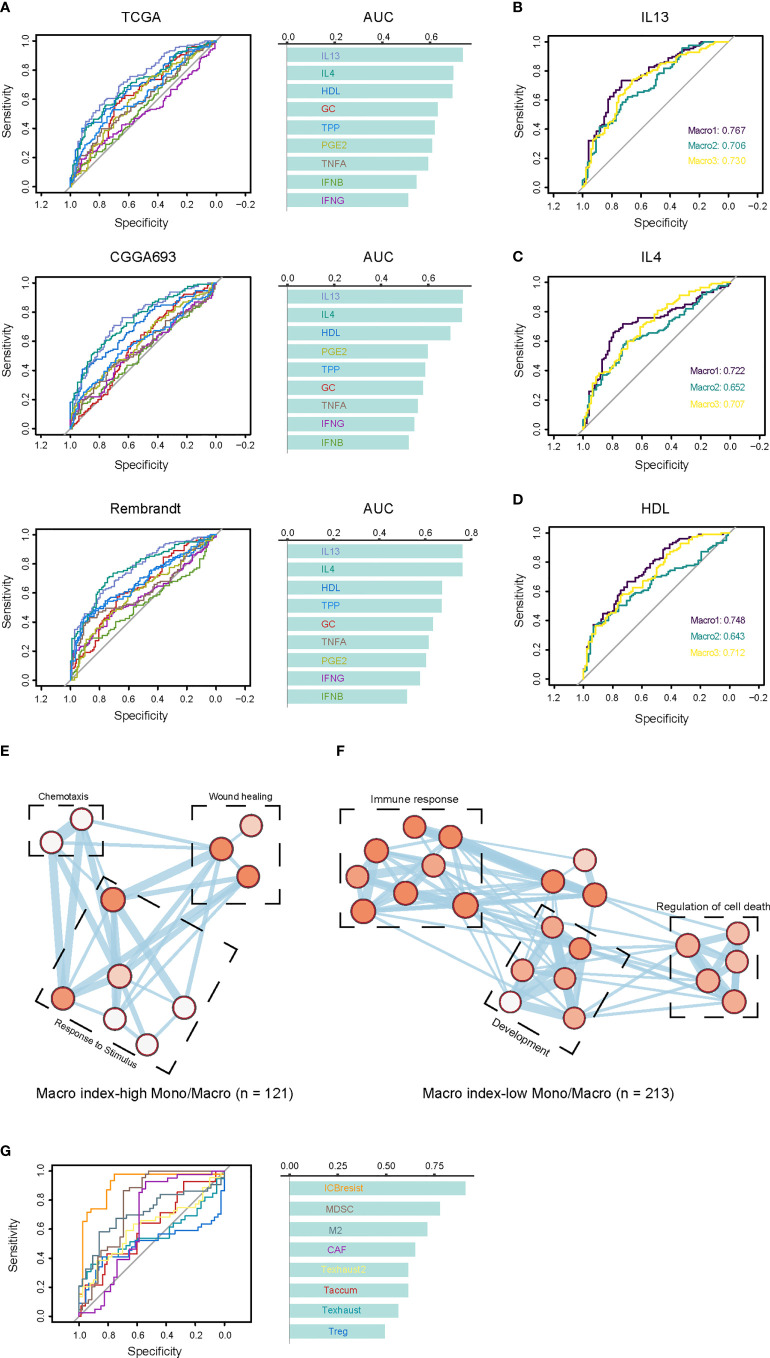
Association between the Macro index and the TAM phenotypes. **(A)** The consistency between the Macro index and gene signatures of TAM phenotype. Cells stimulated with GMCSF for 72 hours were M0 macrophages, and positive cells are cells cultured for 72 hours under different conditions after GMCSF-induced differentiation. Genes defined as positive or negative hits were the top 150 up- or down-regulated in the positive vs. M0 group, respectively. The ROC curves measure the performance of the Macro index in the prediction of the positive or negative hit genes based on the significant Spearman rho of the two. **(B-D)** The efficiency Macro index in predicting positive and negative hit genes induced by IL13, IL4, and HDL in each group. **(E, F)** Enrichment analysis of the biological processes of DEGs in Mono/Macro cells of the Macro index-high and Macro index-low groups. The color of the bubbles is inversely proportional to the q value of the enrichment score. **(G)** The prediction of T cell dysfunction-related gene signatures by the Macro index.

Since the Macro index correlated with the M2 fraction, we proceeded to interrogate its association with the functional status of TAMs. TAMs were loosely defined as M1 and M2 phenotypes, and recent studies have shown the complexity of functional states of macrophages induced and activated by different stimulators (23). We defined the positive and negative hit genes as the top and bottom 150 DEGs of macrophages under each condition (**Supplementary File 1**) and determined whether the Macro index predicted positive or negative hit genes. We found that the macro index performed better in predicting macrophages induced by IL13, IL4, and HDL, with similar efficiency between glioma groups (**Figures 5A-D**). From a single-cell perspective, genes comprised of the Macro index were mainly expressed by monocytes and macrophages (Mono/Macro) in glioma (**Figure S7A**). We extracted the expression profile of monocytes/macrophages and grouped the monocytes/macrophages according to the median Macro index. As a result, the Macro index-high group was mainly transcriptionally regulated by NF-KB and STAT3 (**Figures S7B, C**), corroborating that STAT3 induces the immunosuppressive phenotype of glioma TAMs (55). Besides, the Macro index-high group had increased expression of CD163, as well as other macrophage alternative activation-related gene signatures (**Figure S7D**). Functional enrichment analysis found that genes upregulated in the Macro index-high monocytes/macrophages mainly enriched in BPs including wound healing, chemotaxis, and response to stimulus (**Figure 5E**). Genes upregulated in the Macro index-low group were mainly involved in the inflammatory response (**Figure 5F**). Moreover, recent studies based on the shRNA screen have identified genes involved in T cell dysfunction. Using ROC curves, we found that the Macro index gave the best performance in predicting ICB resistance (anti-CTLA4), as well as MDSC and M2 (**Figure 5G**). Therefore, these results highly suggested that the Macro index was associated with an immunosuppressive M2 phenotype of TAMs, and was involved in the T cell dysfunction.

### The prognostic significance of the Macro index

**Figure 6:**
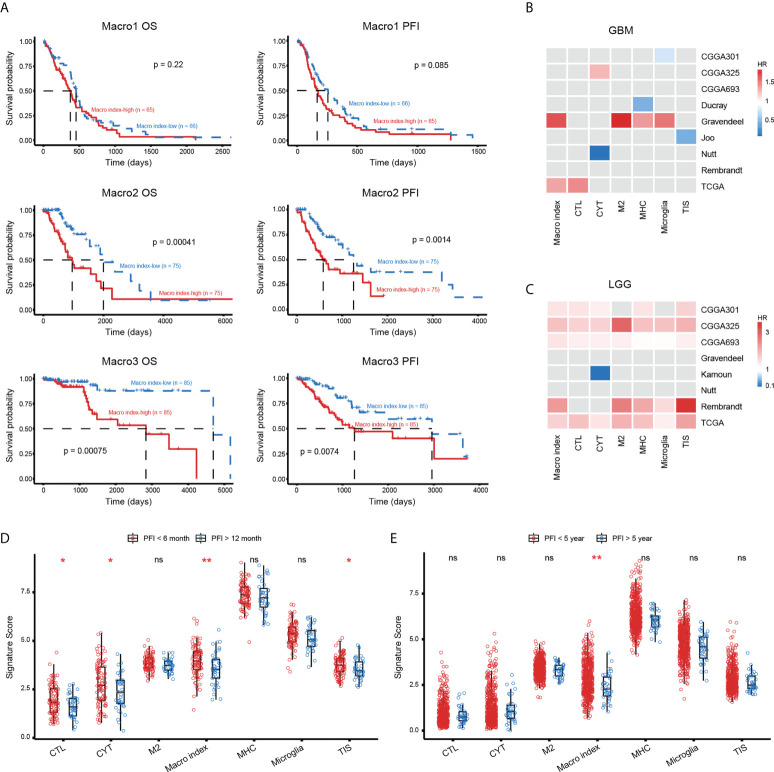
The prognostic significance of Macro index-based groups. **(A)** The differences in OS and PFI between the Macro index-high and -low groups based on the TCGA cohort. (**B**, **C**) The prognostic significance of the Macro index as well as other immune-related gene signatures in GBM and LGG of multiple cohorts. The Gray box represents statistically insignificant. CTL, cytolytic T lymphocyte; CYT, cytolytic activity; MHC, major histocompatibility complex; TIS, T cell inflammation signature. **(D)** Comparison of the scores of immune gene signatures in GBM patients that were early relapsed (PFI < 6 months) and late relapsed (PFI > 12 months). **(E)** Comparison of the scores of immune gene signatures in LGG patients that were early relapsed (PFI < 5 years) and late relapsed (PFI > 5 years). *p < 0.05, **p < 0.01, ns, non significant.

Then, we explored the prognostic significance of the Macro index. As a result, K-M analysis showed that an increased Macro index predicted decreased OS and PFI in both Macro2 and Macro3 (**Figure 6A**), thus an unfavorable outcome in LGG (**Figures S8A-C**). From a broader perspective, the Macro index was a robust risk prognostic factor for LGG, which performs comparably to other immune-related gene sets, but not for GBM (**Figures 6B, C**). In addition, the GBM samples were split into early (PFI < 6 months) and late (PFI > 12 months) relapse groups. Macro index, as well as several other immune-related indicators, such as CTL, CYT, and TIS, were significantly decreased in the late relapse group, suggesting an association between Macro index and disease progression (**Figure 6D**). Notably, the LGG was split into two groups based on the PFI and the Macro index was the only immune-related biomarker that may indicate PFI beyond 5 years in LGG (**Figure 6E**). Further at the protein level, we found that the Macro index was positively correlated with PAI1 (Spearman rho = 0.286, p = 0.015), and GBM patients with decreased PAI1 had prolonged PFI (**Figures S8D, E**). Together, these results indicate that the Macro index was a robust prognostic biomarker for LGG, and its clinical implication in GBM needs further exploration.

### The Macro index was associated with ICI responsiveness

**Figure 7:**
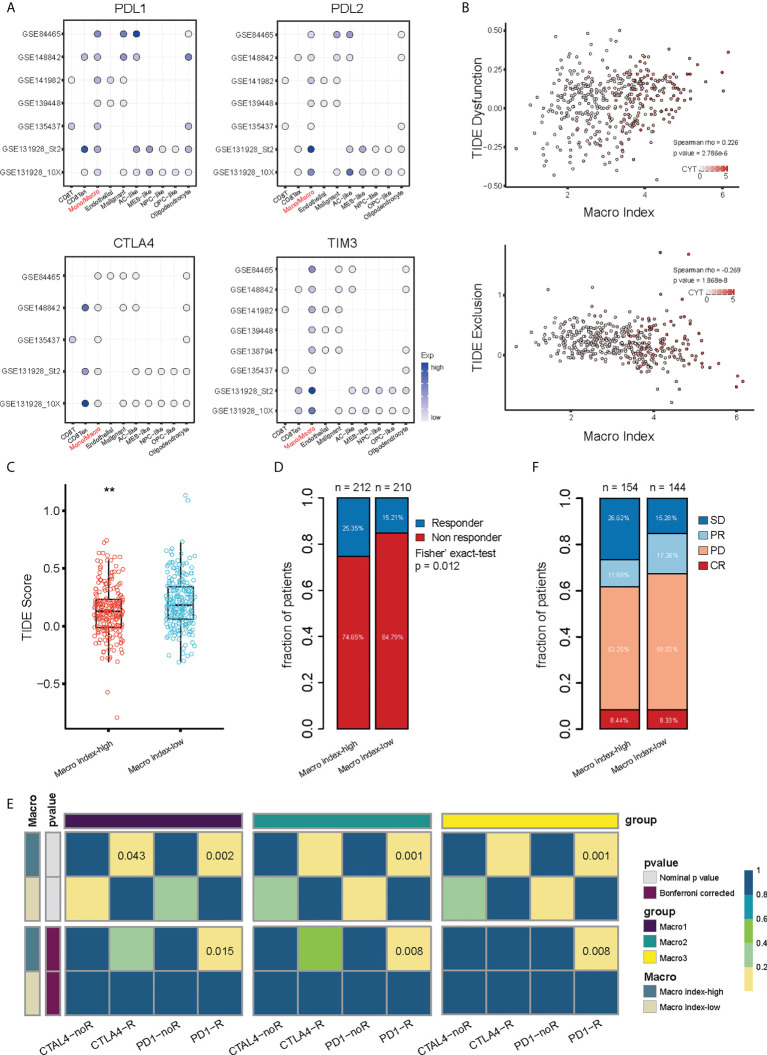
Association between the Macro index and ICI responsiveness. **(A)** Expression of immune checkpoints by different types of cells in the glioma TME at the single cell scope. **(B)** Association between the Macro index, TIDE T cell Dysfunction score, TIDE T cell Exclusion score, and CYT. Spearman rho measures the correlation between Macro index with Dysfunction and Exclusion scores. **(C)** TIDE score between Macro index-high and -low groups of TCGA glioma. **(D)** Distribution of predicted ICI responders and non-responders between the Macro index-high and -low groups. **(E)** Sample responsiveness to anti-PD1 or anti-CTLA4 was evaluated using the unsupervised Submap algorithm. **(F)** Macro index-based group in predicting the ICI benefit of the uroepithelial carcinoma sample. SD, stable disease; PR, partial response; PD, progression disease; CR, complete response. **p < 0.01.

M2 macrophages are vital in remodeling the TME and frustrating the anti-tumor immune response (56, 57), therefore, we investigated the relationship between the Macro index and the immune checkpoint blockade that aimed at reviving the antitumor immune response by relieving the inhibition of cytolytic T lymphocyte by TME. Integration of multiple glioma single-cell expression profiles revealed that considerable immune checkpoints were expressed by monocytes/macrophages in the glioma TME (**Figure 7A**), which suggested that TAMs in the glioma TME may impede the anti-tumor immune response through immune checkpoints. TIDE provides a computational framework for assessing tumor immune evasion, i.e., induction of T-cell dysfunction in tumors with high CTL infiltration and prevention of T-cell infiltration in tumors with low CTL infiltration (51). Evidence suggested that gliomas, especially LGG, have little T-cell infiltration and abundant TAMs and are therefore lymphocyte-depleted or immune-quiet tumors (58). In our context, the Macro index was positively correlated with T-cell dysfunction and negatively correlated with T-cell exclusion (**Figure S9**), indicating that induction of T-cell dysfunction was the predominant mode of immune evasion in samples with an increased Macro index. Consistently, samples with elevated Macro index and TIDE T-cell dysfunction scores scored higher in cytolytic activity (**Figure 7B**), and the Macro index-high group had decreased TIDE score (**Figure 7C**), characterizing higher levels of antitumor activity with lower levels of tumor immune evasion. Notably, TIDE predicted that a greater proportion of patients in the Macro index-high group were likely to respond to ICI (fisher’s exact p = 0.012) (**Figure 7D**). Although we have shown that increased Macro index was strongly associated with anti-CTLA4 resistance, unsupervised machine learning algorithms suggested that these samples may still benefit from anti-PD-1 treatment (**Figure 7E**). As an indirect testimony to the significance of the Macro index in facilitating the application of ICI therapy, a similar approach was employed to stratify the uroepithelial carcinoma cohort that received ICI treatment, and the proportion of patients with stable disease (SD) after anti-PD1 therapy was significantly higher in the Macro index-high group (fisher’s exact test p = 0.023) (**Figure 7F**). Taken together, these results indicated that the Macro index showed promise for facilitating the application of PD-1 antibodies in glioma, which deserves further investigation.

## Discussion

The importance of TAMs in the glioma TME cannot be overstated. With the rise of ICI therapy, there has been an increasing interest in the immunological properties of glioma TME (57). TAMs are involved in the failure of the anti-tumor immune response by promoting the formation of an immunosuppressive TME (15, 16), which makes them one of the sizzling therapeutic targets. Our knowledge of glioma TAMs remained at the M2 phenotype, a category that encompasses several functional states, which has hindered the development of TAMs-targeting therapies. Furthermore, recent studies have found that the metabolic and functional states of immune cells are conjugated and that alterations in lipid metabolism have important implications for the TME (24–26, 59). This has inspired the exploration of the association between the lipid metabolism of TAMs and the immunological properties of the glioma TME.

Several studies highlighted the impact of the PI3K/Akt pathway on macrophages. For example, advanced oxidative protein products inhibit autophagy by activating the PI3K/AKT/mTOR pathway, leading to macrophage dysfunction and impaired M1 polarization (60). Also, PM2.5 activates macrophages in a PI3K/Akt signaling-dependent manner. Akt is essential for the IL-4-induced M2 polarization of macrophages and the deficiency of TSC attenuates such a program by regulating mTOR (61). Particularly, TSC-deficient bone marrow-derived macrophages were impaired in the induction of Arg1, Fizz1, and Ym1 by IL-4 (62). Therefore, the PI3K/Akt signaling plays a role in macrophage M2 polarization. The PI3K/Akt signaling pathway also induces increased levels of FA β-oxidation in M2-type macrophages under chronic inflammatory conditions, thereby maintaining sustained energy expenditure (26). However, TAMs in the GBM TME may not have increased mitochondrial β-oxidation compared to LGG, as we found the expression of CPT1A/B/C was significantly reduced in Macro1. Furthermore, the relatively reduced FA β-oxidation in macrophages of the glioma TME may lead to the greater shunt of FA to phospholipids and then AA, the substrate of eicosanoids, and overexpression of lipid carriers should also allow for a greater influx of exogenous FAs or AA, making macrophages to be vehicles for tumor shaping of TME.

The interaction between PI3K/Akt signaling and eicosanoids is less characterized. Tong WG et al. reported that LTB_4_ activates PI3K/Akt signaling and the blockade of the PI3K pathway using wortmannin attenuated LTB_4_-mediated tumor cell proliferation (63). Likewise, PGE_2_ promotes tumor cell invasion and metastasis in a PI3K/Akt-dependent manner (64). Therefore, it appears that eicosanoid derivatives promote various malignant behaviors of tumor cells *via* the PI3K/Akt signaling pathway. Nevertheless, insufficient evidence is found for direct regulation of LTs production by the PI3K/Akt pathway. Nikos Koundouros et al. demonstrated that mutant PIK3CA facilitates the production of AA and subsequently eicosanoids through activating PI3K/Akt/PLA2 axis (65). Zhou et al. also reported an association between the PI3K/Akt signaling pathway and the expression of ALOX5 in breast cancer (66). Mechanistically, PI3K/Akt signaling regulates the activity of multiple FA synthesis and transport enzymes, thereby funding anabolism (26, 27). PLA_2_ is the main enzyme that dissociates AA from phospholipids when cells encounter a stimulus resulting in increased intracellular Ca^2+^ (67). Gimenes et al. reported that γCdcPLI1 inhibits the activity of PLA_2_ in a PI3K/Akt dependent manner, possibly by interfering with the expression of Akt1/3 and PI3KR1 (68). Besides, PI3K also funds the activity of Crotoxin B, a catalytically active subunit IIA sPLA (69). These results are not yet sufficient to demonstrate a regulatory relationship between the PI3K signaling pathway and leukotriene production, and we raise the possibility by correlating the PI3K signaling pathway with the alternative activation of TAMs and eicosanoid metabolism.

The Macro index comprised of PIK3R5, PIK3R6, ALOX5, ALOX5AP, and ALOX15B serves as a valid prognostic biomarker for gliomas, especially LGG. Macro index is not a valid prognostic predictor for GBM, although abnormalities in the PI3K signaling pathway are present in over half of GBM (70). This may be related to the consensus that it is mainly the deletion of PTEN or abnormal activation of RTKs that accelerates the development of GBM. Although PIK3R5 and PIK3R6 are involved in the encoding of regulatory subunit of the class I PI3K gamma complex, their weight in the Macro index is diluted by three other genes related to leukotriene synthesis, whose impact on the GBM microenvironment is not yet known. Interestingly, we did not directly screen for genes of prognostic value, thus these results preliminarily confirmed the important role of the PI3K/Akt pathway and leukotrienes in glioma. Notably, we found that the Macro index-high group expressed more immune checkpoints and was characterized by dysfunction of CD8 T cells. Decreased TIDE scores in the Macro index-high group may indicate reduced levels of CD8 T cell dysfunction as well as immune evasion, as we have shown that the Macro index was positively correlated with T cell dysfunction score. Overall, Macro index acts as an inflammatory biomarker of the glioma microenvironment and is associated with the recruitment of multiple immune cells. However, M2-type polarization of macrophages and release of leukotrienes in this type of TME hindered the function of effector T cells, which may be one of the reasons that the Macro index is associated with ICI responsiveness in gliomas. Therefore, leukotriene synthesis and alternative activation of TAMs characterized by the Macro index are essential for the regulation of immunity in glioma TME where various factors intermingle.

## Data availability statement

The original contributions presented in the study are included in the article/**Supplementary Material**. Further inquiries can be directed to the corresponding authors.

## Author Contributions

SH, HJ, ZL, and JJ conceived and designed the study. HJ, HZ, XY, and NW were responsible for data collection, curation, quality control, and analysis. ZL and JZ reviewed and repeated the process of data analysis. HJ and ZL wrote the original draft of the manuscript, which was reviewed and revised by QG, JD, and FW. HJ and HS drafted and typeset the figures. CY, SH, and YL supervised the study. All authors contributed to the article and approved the submitted version.

## Funding

This work was funded by the National Natural Science Foundation of China (No. 61575058) and Zhejiang Provincial People’s Hospital Talent Introduction Project (No. C-2021-QDJJ03-01).

## Acknowledgments

The authors gratefully acknowledge databases including TCGA, CGGA, UCSC, GlioVis, TISCH, and many other open access data portals for offering convenient access to datasets and user-friendly online analysis.

## Conflict of interest

The authors declare that the research was conducted in the absence of any commercial or financial relationships that could be construed as a potential conflict of interest.

## Publisher’s note

All claims expressed in this article are solely those of the authors and do not necessarily represent those of their affiliated organizations, or those of the publisher, the editors and the reviewers. Any product that may be evaluated in this article, or claim that may be made by its manufacturer, is not guaranteed or endorsed by the publisher.
