# Supplementary material for: Integrated genomic, transcriptomic, and epigenetic analyses identify a leukotriene synthesis-related M2 macrophage gene signature that predicts prognosis and treatment vulnerability in gliomas
Source: Front Immunol. 2022 Sep 8;13:970702. doi: 10.3389/fimmu.2022.970702 (PMC9493442; doi:10.3389/fimmu.2022.970702)
Supplement: Supplementary file 1 [file DataSheet_1.docx]

Supplementary Material

# Supplementary Figures


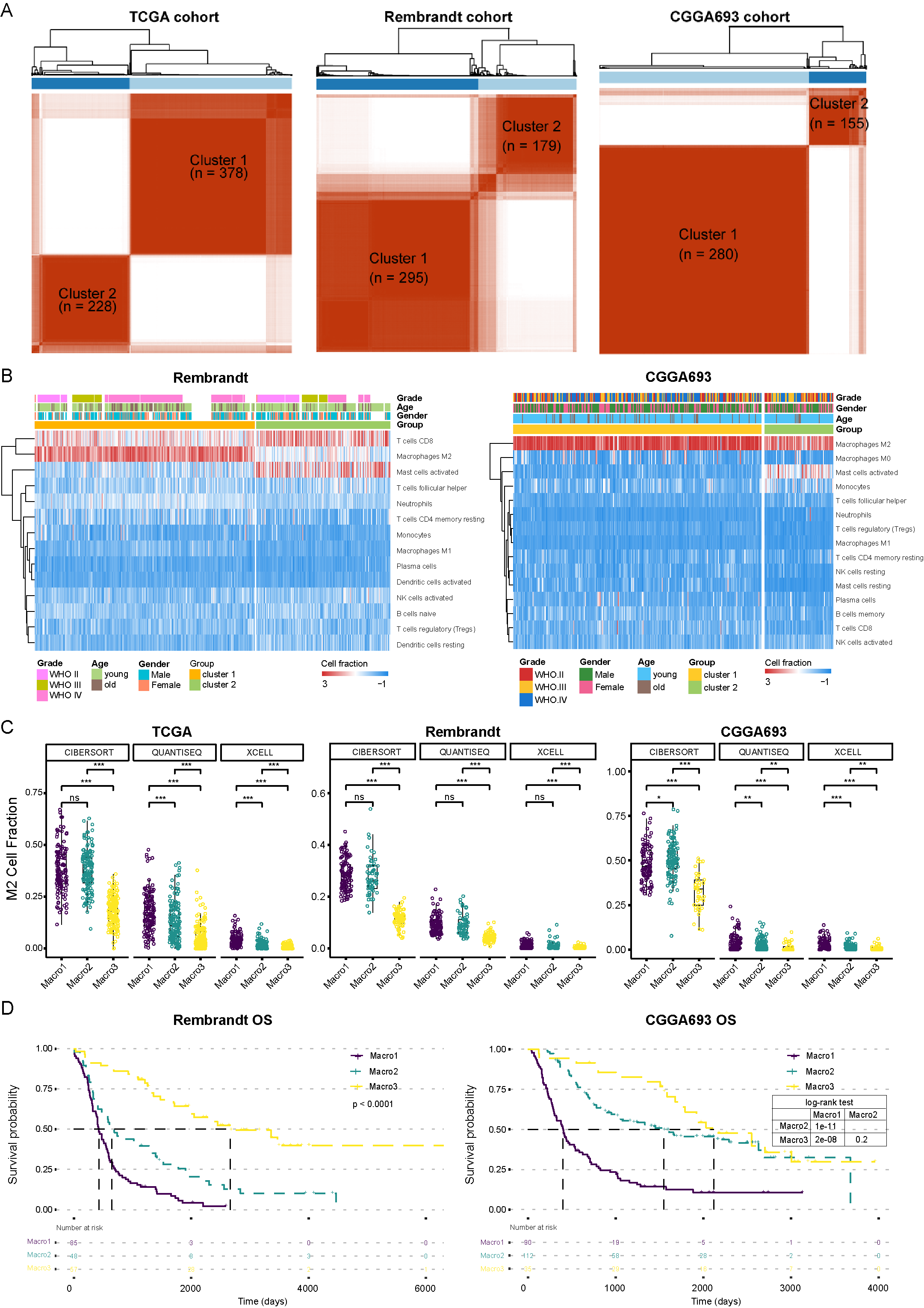


**Supplementary figure 1. Classification of glioma samples.** (**A**) Consensus clustering based on immune infiltration calculated by CIBERSORT. (**B**) Distribution of immune infiltration in different clusters. Immune cells with 0 fractions over half of the samples were excluded. (**C**) Comparison of M2 macrophage fractions among glioma groups. (**D**) Survival differences between glioma groups using external validation cohorts.


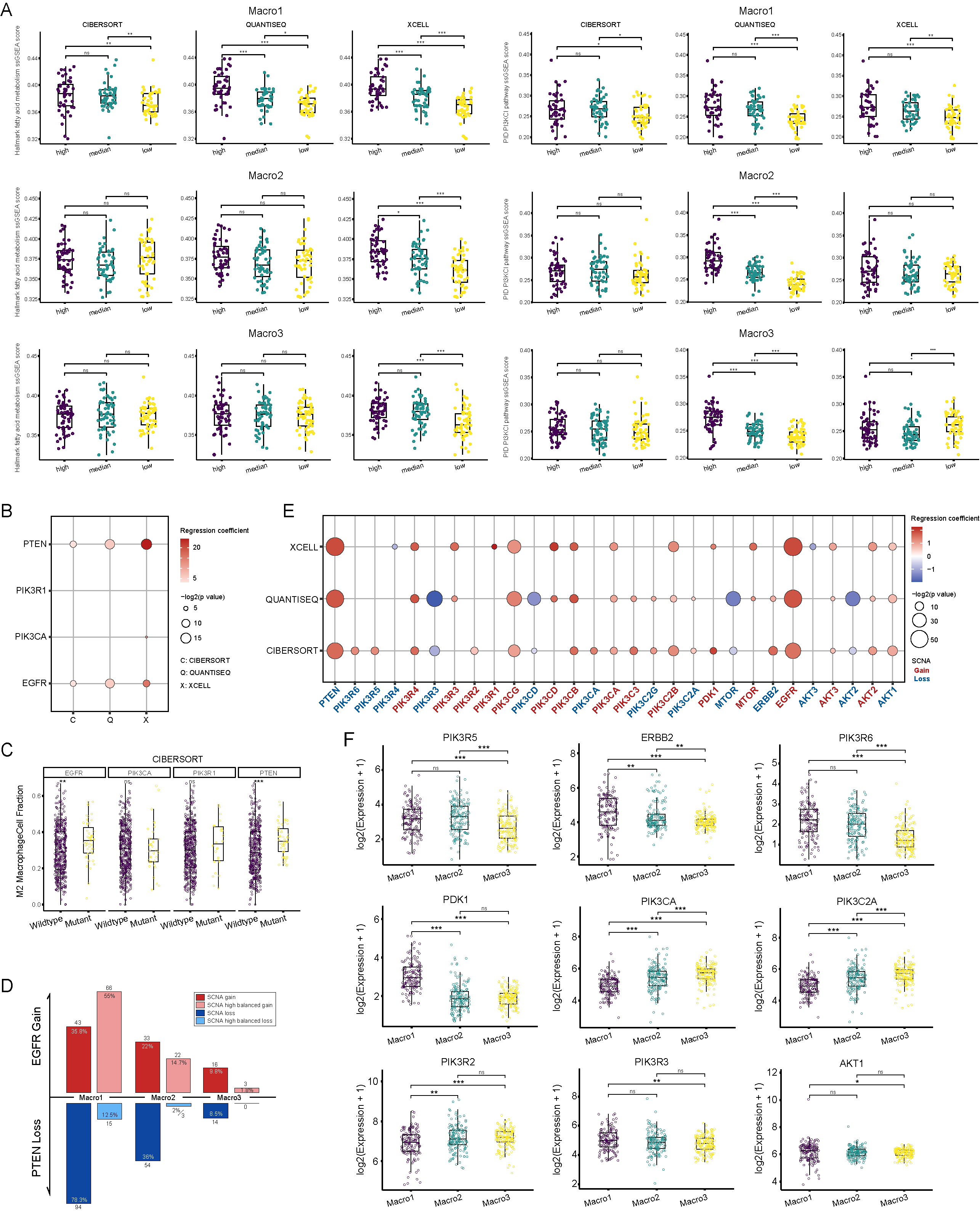


**Supplementary figure 2. Association of M2 fraction with the PI3K pathway and FA metabolism.** (**A**) To validate the association of the M2 fraction with the PI3K pathway and FA metabolism, glioma samples were divided into three groups based on the M2 fraction: M2-high, M2-median, and M2-low. The ssGSEA score of the two pathways between groups was compared. (**B**, **C**) Correlation between PI3K pathway members with M2 fraction. Logistic regression analysis was employed to evaluate the regression coefficient. (**D**) Distribution of the SCNA frequency of EGFR and PTEN. (**E**) Estimating the association between the SCNA of PI3K pathway members and M2 fraction using logistic regression analysis. (**F**) Expression of genes of interest.


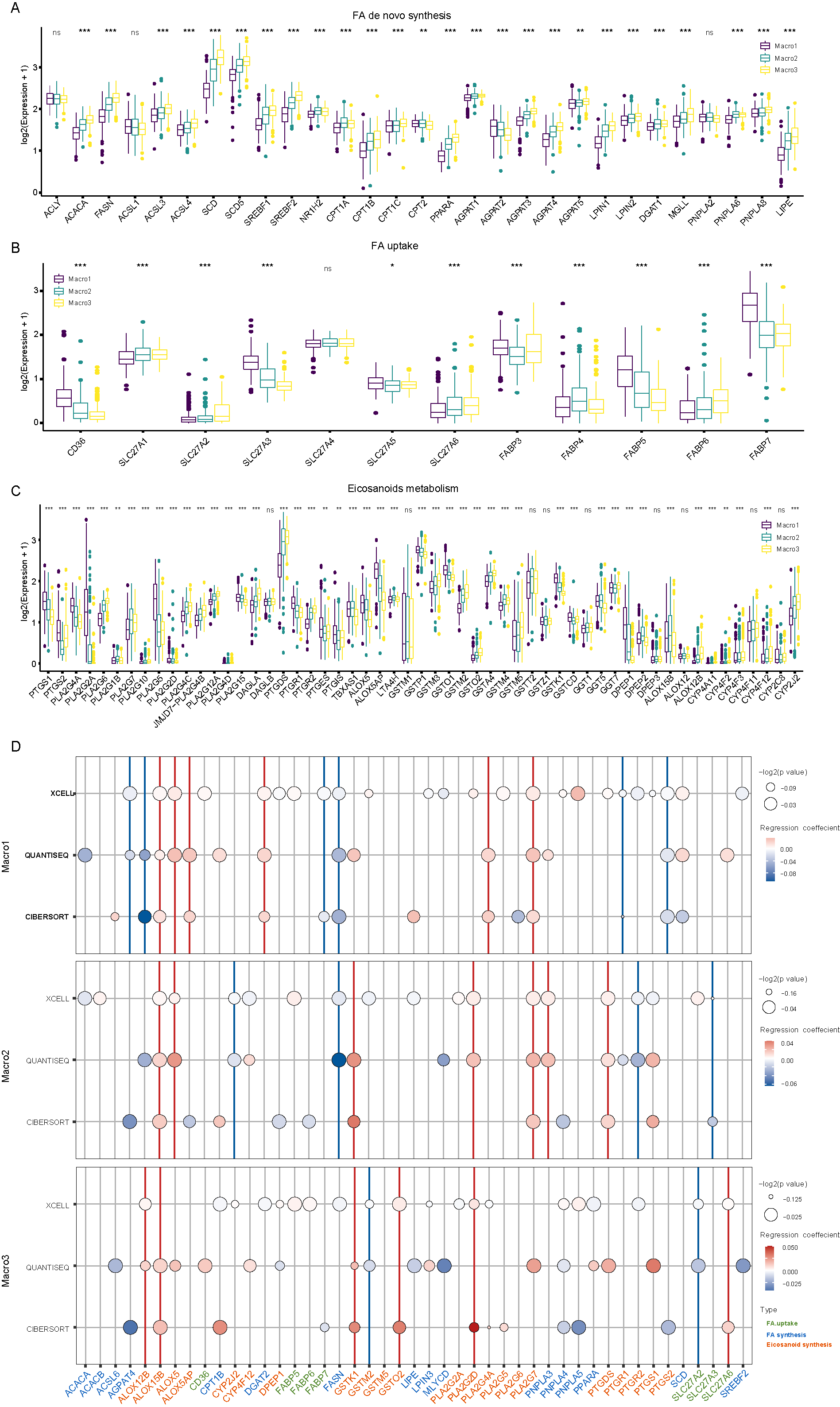


**Supplementary figure 3. Expression of FA metabolism-related genes and their association with M2 fraction.** (**A**-**C**) Expression of genes involved in the FA de novo synthesis, FA uptake, and eicosanoid metabolism between glioma groups. (**D**) Estimating the association between FA metabolic-related genes and M2 fraction based on multivariate linear regression. We determine two or more regression coefficients that are significant and have consistent positive or negative signs as statistically significant and use the red vertical line to indicate a positive correlation and blue to indicate a negative correlation.


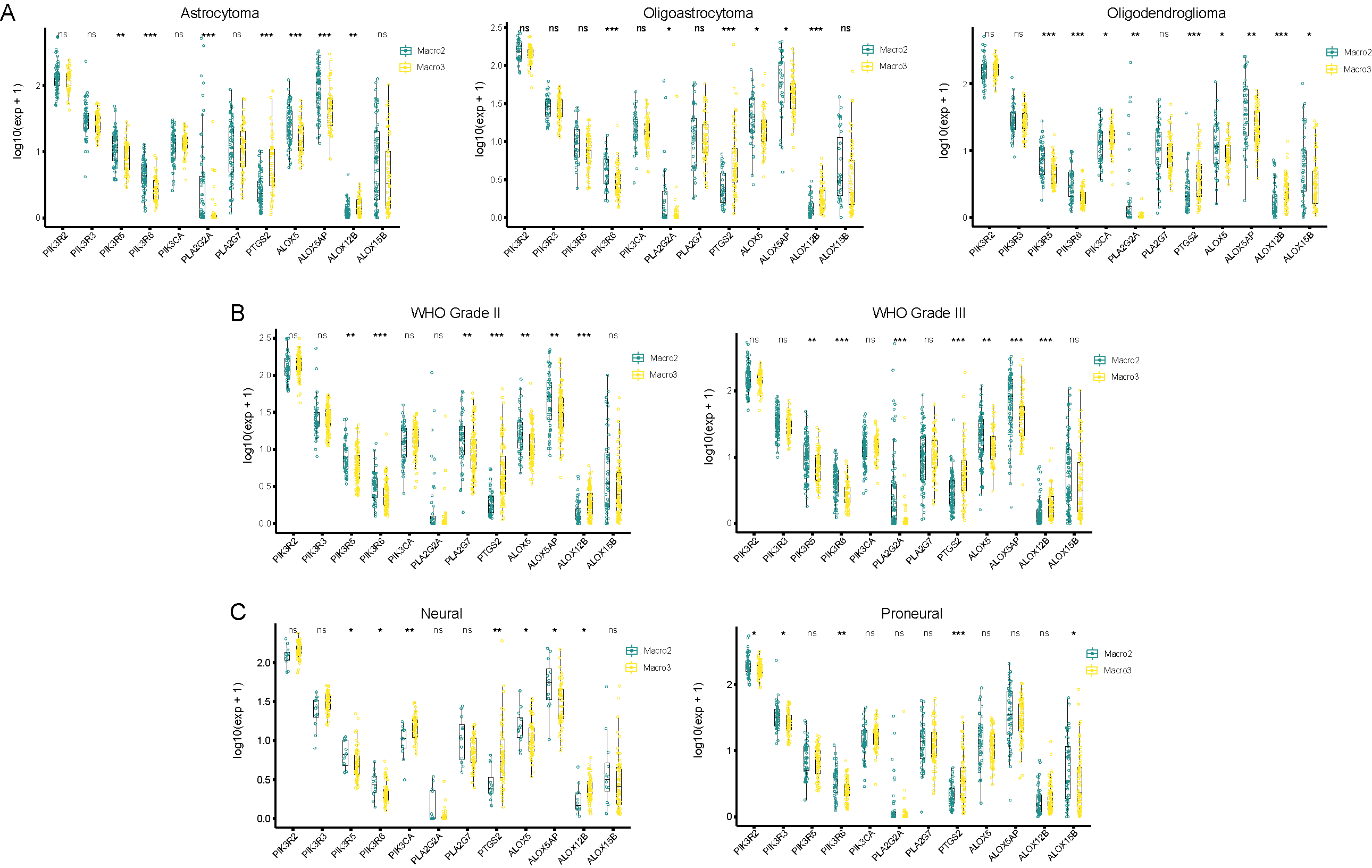


**Supplementary** **figure 4. Expression of PI3K pathway members and FA metabolism genes of interest in Macro2 and Macro3.** Histological (**A**), WHO tumor grade (**B**), and transcriptome subtype (**C**) were corrected sequentially.


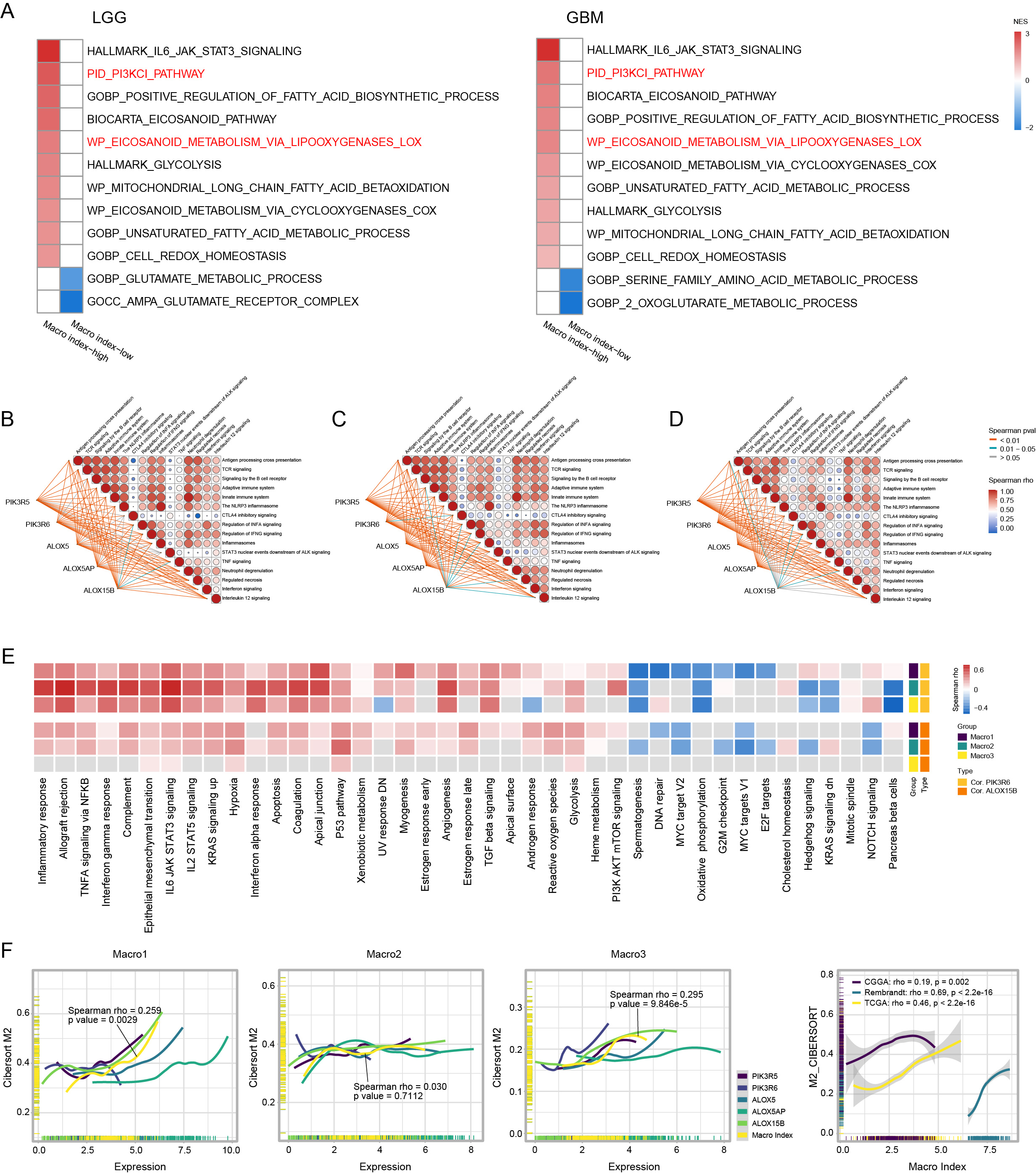


**Supplementary figure 5. Molecular underpinnings of the Macro index.** (**A**) GSEA analysis reveals the signaling pathways enriched in the Macro index-high and -low groups in GBM and LGG. (**B**-**D**) Differential correlation of genes comprising the Macro index with KEGG lipid metabolism-related signaling pathway. (**E**) The association of PIK3R6 and ALOX15B with the HALLMARK gene sets between groups. (**F**) Correlation of Macro index and genes constituting Macro index with M2 fraction, and the association between the Macro index with M2 fraction estimated using CIBERSORT in multiple cohorts.


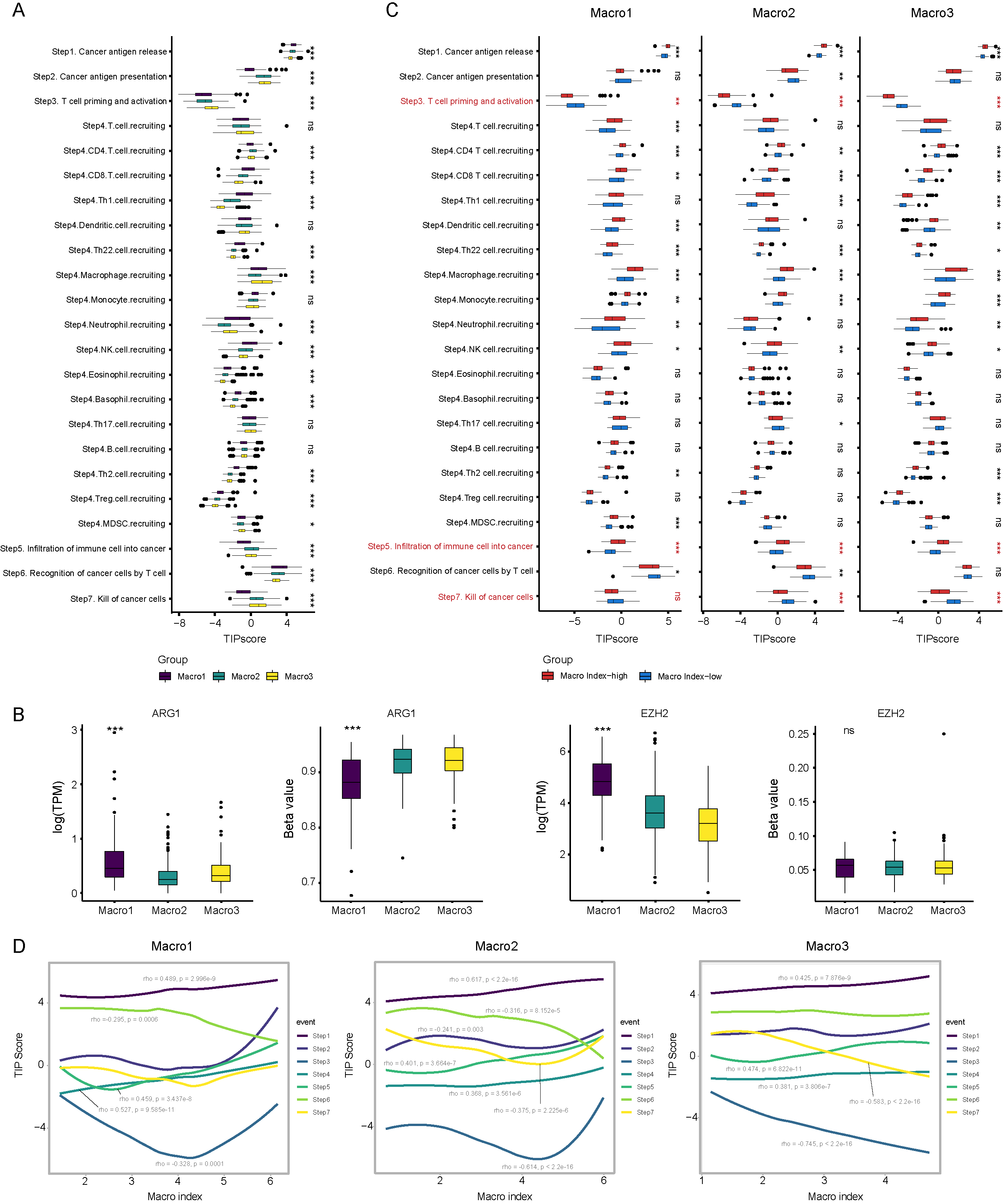


**Supplementary figure 6. Correlation of the Macro index with anti-tumor immune response.** (**A**) Comparison of the activity of each stepwise event of the antitumor immune response among glioma groups. (**B**) The expression and methylation levels of ARG1 and EZH2 between glioma groups. Although the expression levels of many genes are colocalized with their methylation levels, some exceptions exist, e.g., the expression of EZH2 is associated with Macro index rather than methylation. (**C**) The activity of each stepwise event of the antitumor immune response among Macro index-high and -low groups in each glioma group. (**D**) Correlation between the Macro index with each stepwise event of the antitumor immune response.


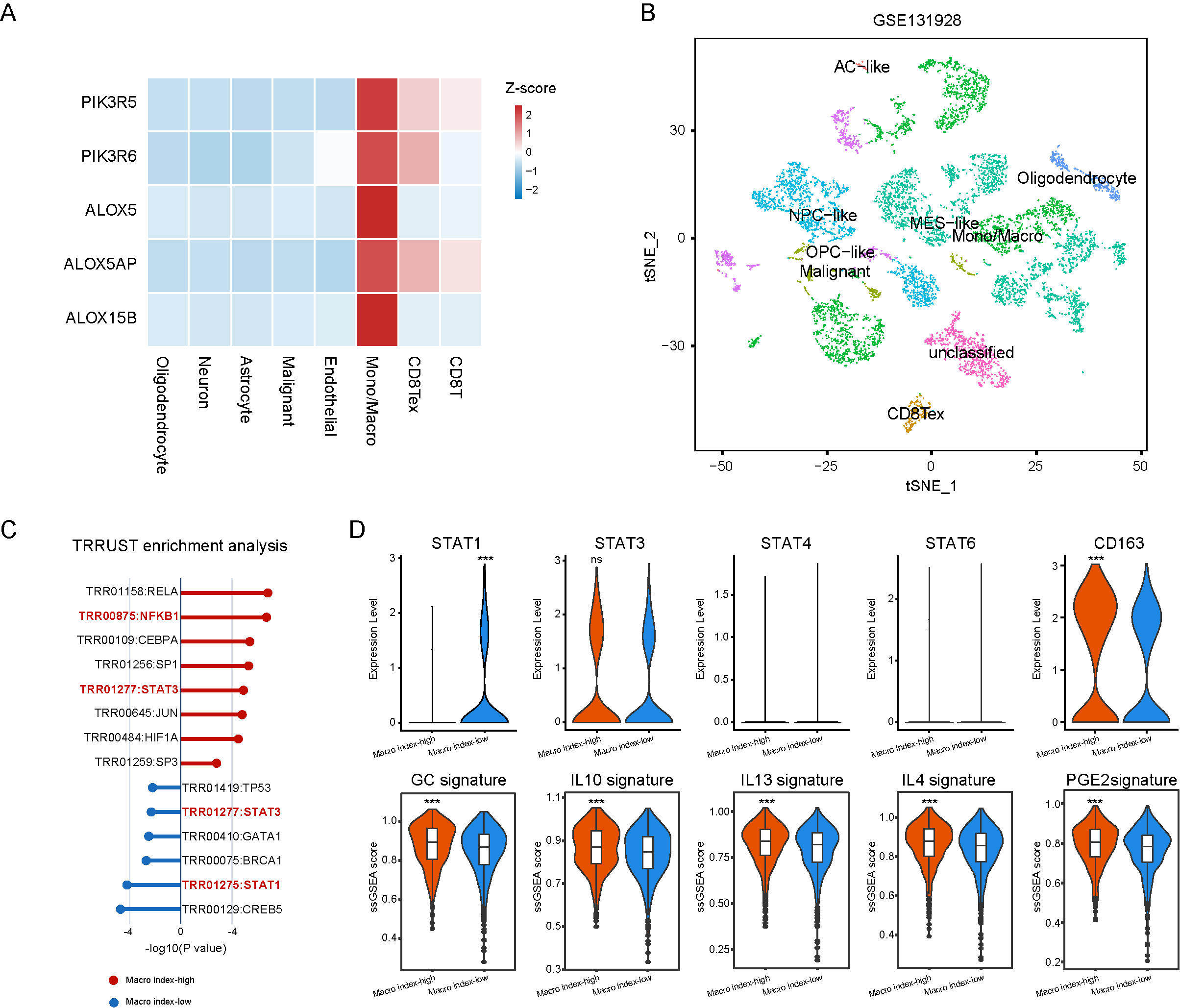


**Supplementary figure 7. Association of Macro index with the functional status of TAMs.** (**A**) Expression of genes comprising the Macro index at the single-cell scope. (**B**) Identification of cellular subtypes in the single cell transcriptome. (**C**) We extracted the expression profile of the Mono/Macro subcluster in the single-cell transcriptome, calculated the Macro index of the Mono/Macro subcluster, and classified the Mono/Macro cells into Macro index-high and -low groups, accordingly. We calculated the DEGs (Macro index-high vs. -low) between the Macro index-high and -low groups and performed the functional enrichment analysis. Transcriptional factors of interest were marked red. (**D**) Expression of genes and scoring of gene sets associated with alternative activation of TAMs. Gene sets associated with GC, IL10, IL13, IL4, and PGE2 were defined as the top 50 upregulated genes of macrophages activated by corresponding stimulators.


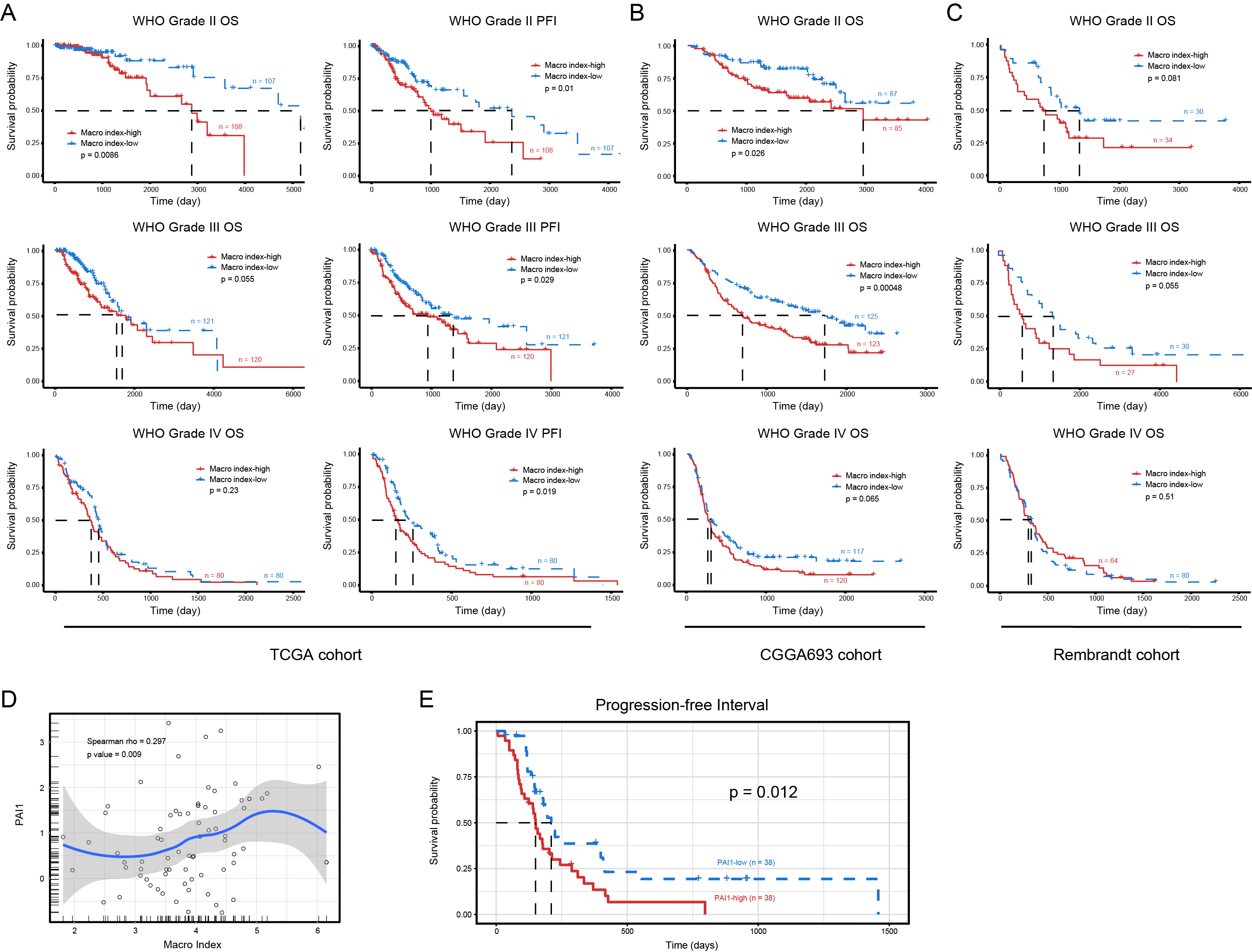


**Supplementary figure 8. The prognostic significance of Macro index-based groups.** Samples were divided by WHO tumor grade. (**A**) TCGA cohort, (**B**) CGGA693 cohort, and (**C**) Rembrandt cohort. As our study was mainly based on the mRNA level, which is not currently a routine clinical practice, we also explored the alterations associated with the Macro index at the protein level using the RPPA data provided by the UCSC database. We found a significant correlation between Macro index and PAI1 (D), and an increase in the latter implied a worse prognosis for GBM patients (E).


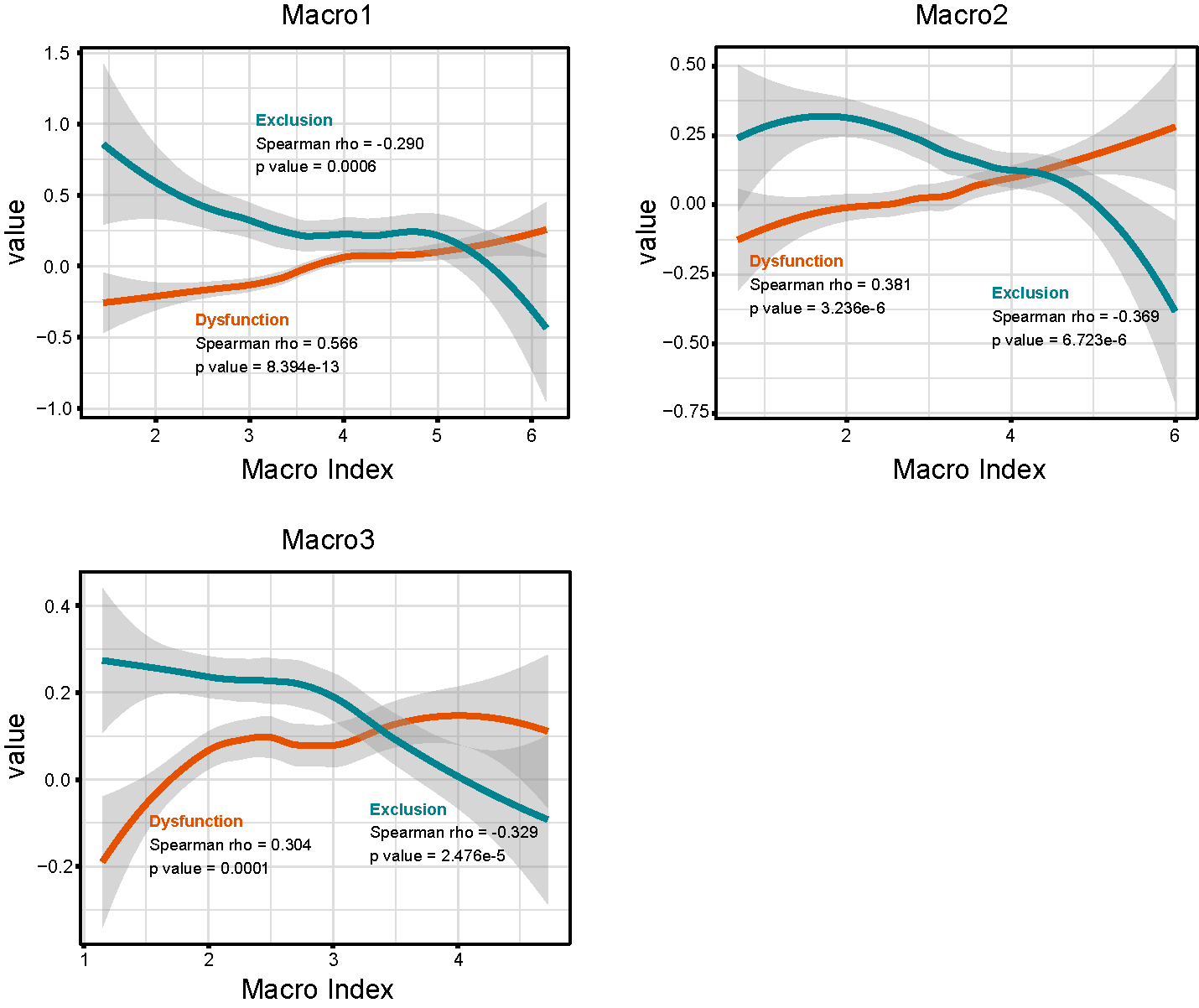


**Supplementary figure 9. The association between the Macro index and tumor immune evasion.** TIDE algorithm was employed to estimate the activity of the 2 types of tumor immune evasion. (**A**) Macro1, (**B**) Macro2, (**C**) Macro3.
